# Supplementary material for: Deconstructing the modular organization and real-time dynamics of mammalian spinal locomotor networks
Source: Nat Commun. 2023 Feb 16;14:873. doi: 10.1038/s41467-023-36587-w (PMC9935527; doi:10.1038/s41467-023-36587-w)
Supplement: Supplementary file 1 — Supplementary Information [file 41467_2023_36587_MOESM1_ESM.pdf]

Supplementary information

Deconstructing the modular organization and real-time dynamics of mammalian spinal locomotor networks

Li-Ju Hsu[1,2], **Maëlle Bertho**[1,2], and **Ole Kiehn**[1,2,*]

[1]Department of Neuroscience, University of Copenhagen, 2200 Copenhagen, Denmark
[2]Department of Neuroscience, Karolinska Institutet, 171 77 Stockholm, Sweden

*Correspondence: Ole.Kiehn@sund.ku.dk

Contents

- **Supplementary Figures 1 to 10**

Table of contents

- Supplementary Fig. 1 .... Page 3
- Supplementary Fig. 2 .... Page 4
- Supplementary Fig. 3 .... Page 5
- Supplementary Fig. 4 .... Page 6
- Supplementary Fig. 5 .... Page 7
- Supplementary Fig. 6 .... Page 8
- Supplementary Fig. 7 .... Page 9
- Supplementary Fig. 8 .... Page 10
- Supplementary Fig. 9 .... Page 11
- Supplementary Fig. 10 .... Page 12

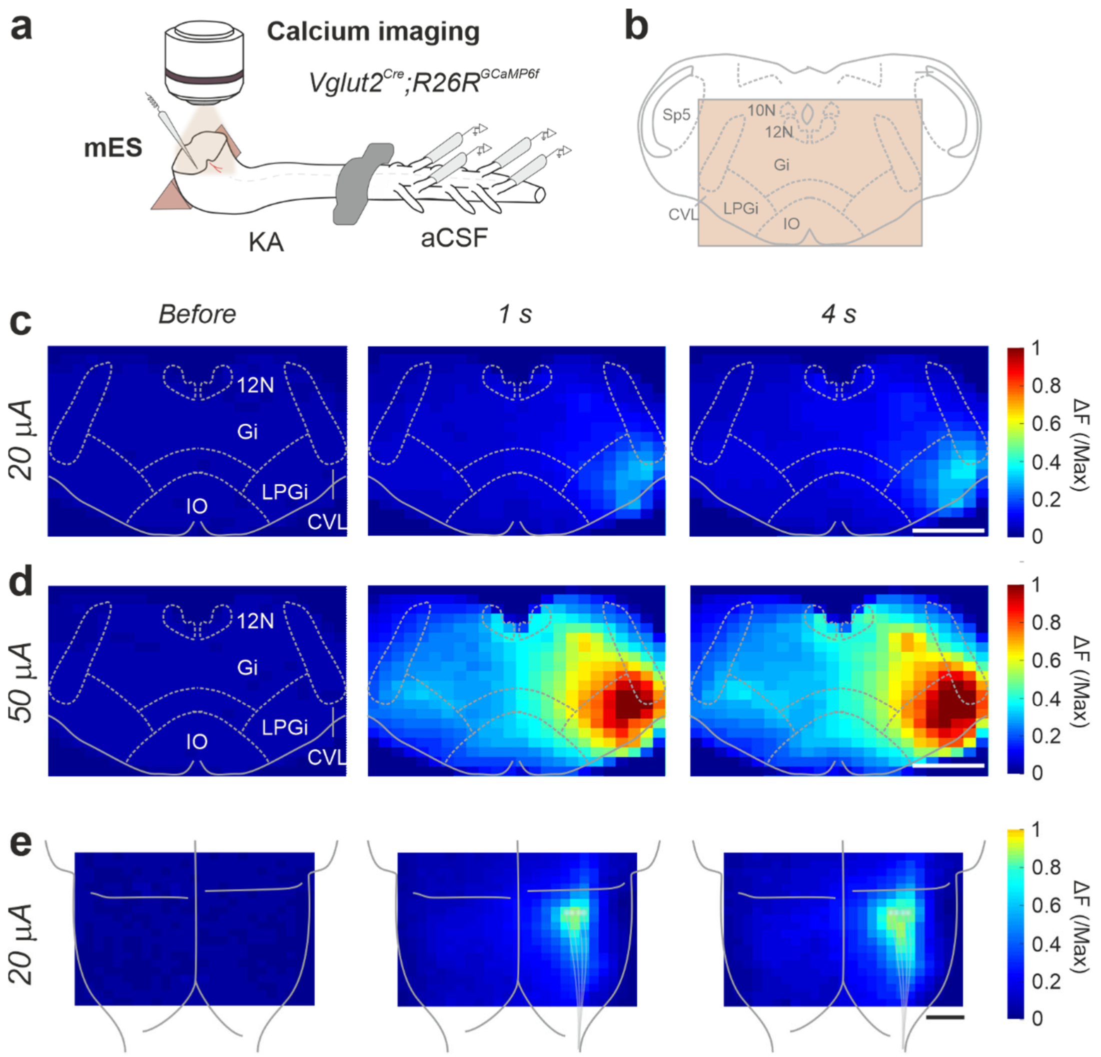

Supplementary Fig. 1 | Brainstem activated area by mES. a Experimental setup. Ca^{2+} imaging of the micro electrical stimulated (mES) LPGi/CVL area in the brainstem in a *Vglut2^{Cre};R26R^{GCaMP6f}* mouse. Drawing adapted from Bouvier et al. 2015. Copyright Elsevier. **b** Imaged area in the transverse brainstem section. The brown square indicates the imaged area. **c-d** Example of mES-activated LPGi/CVL area at low stimulation strength (20 µA, **c**) as compared to activated area of stronger stimulation (50µA, **d**). **e** Example of imaging of the ventral surface of the brainstem showing the mediolateral range of the mES-activated area after stimulation in LPGi/CVL. The ventral extension is the calcium activity in descending axons of the activated neurons.

Scale bars (in µm): (c,d,e): 500

a

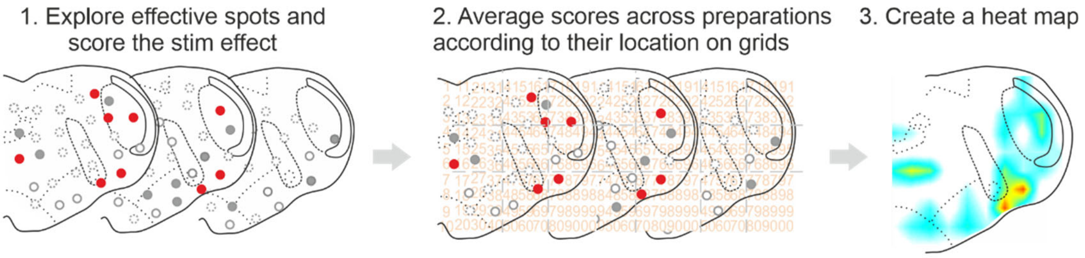

b

Locomotor index	*Score*
● Bursts in 3-4 / 4 VRs with R L alternation	3
● Non-alternated bursts in 3-4 / 4 VRs	2
○ Bursts in 1-2 / 4 VRs	1
◌ No bursts	0

c

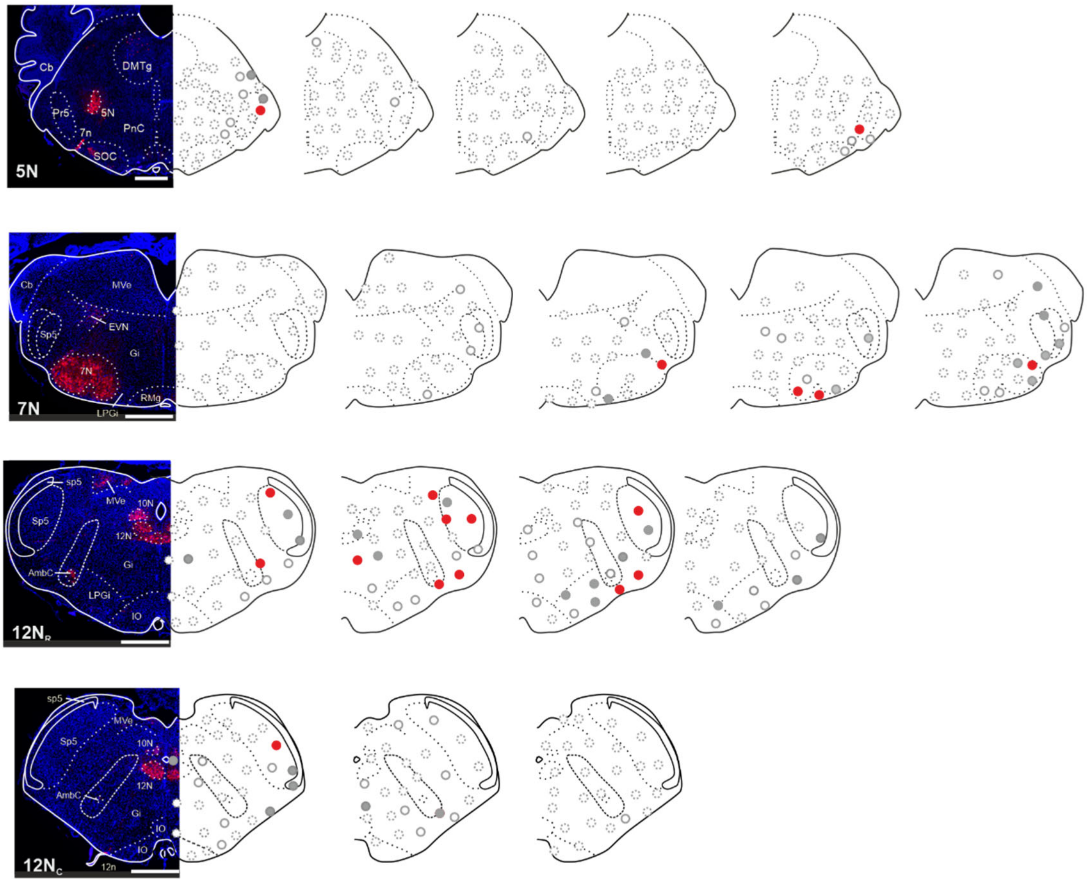

Supplementary Fig. 2 | Effect of mES on specific sites in the brainstem . a Strategies of mapping mES effective spots on the entire transverse section of brainstem. **b** Four locomotor indexes to score the quality of evoked locomotor-like activity. **c** The maps of individual preparations showing the mES-effect on specific sites. Abbreviation as in Figure 1.

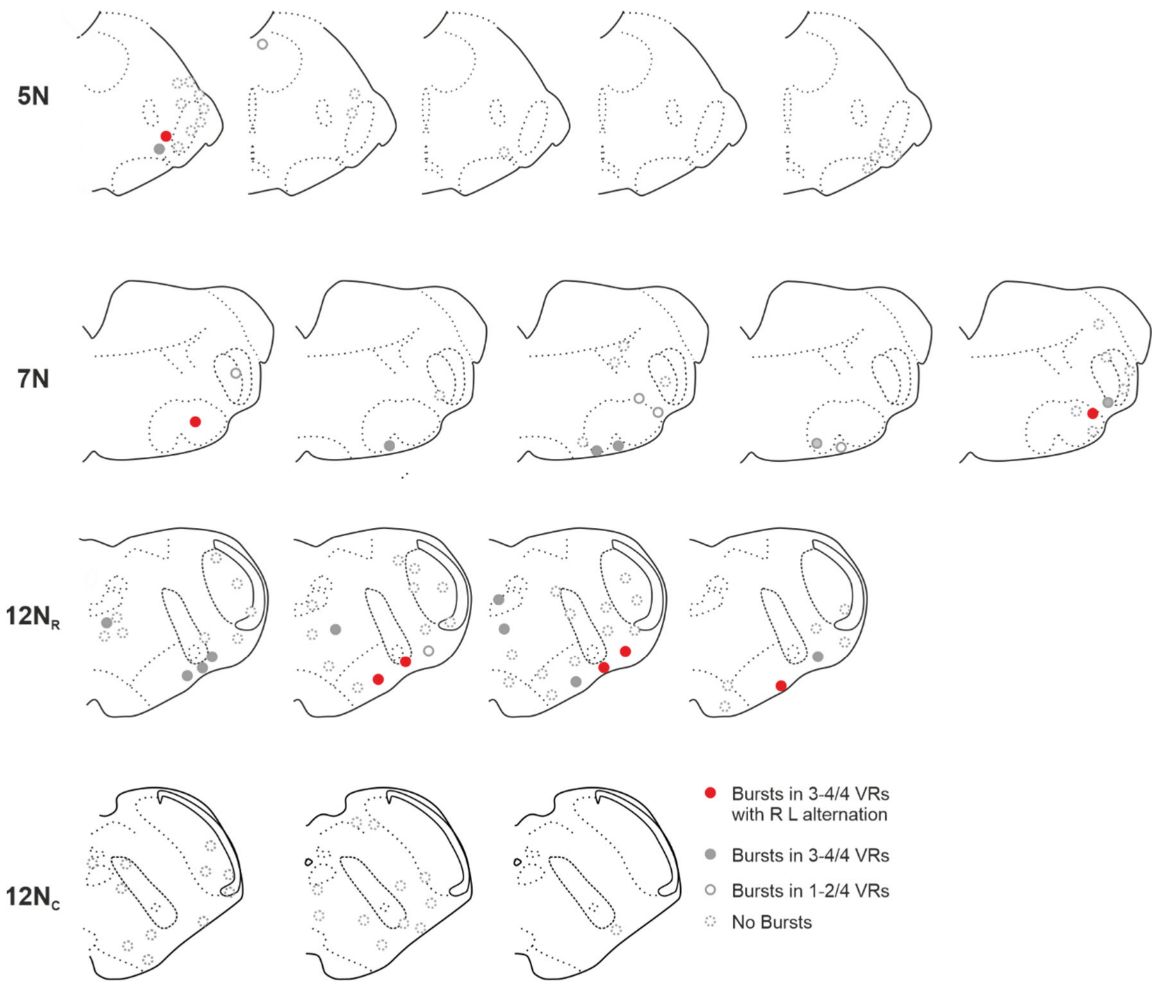

Supplementary Fig. 3 | Neurons located in LPGi/CVL at the rostral 12 N level send a final command to spinal circuits to evoke locomotor-like activity. The maps of individual preparations showing efficient mES sites, with kynurenic acid applied in the brainstem pool.

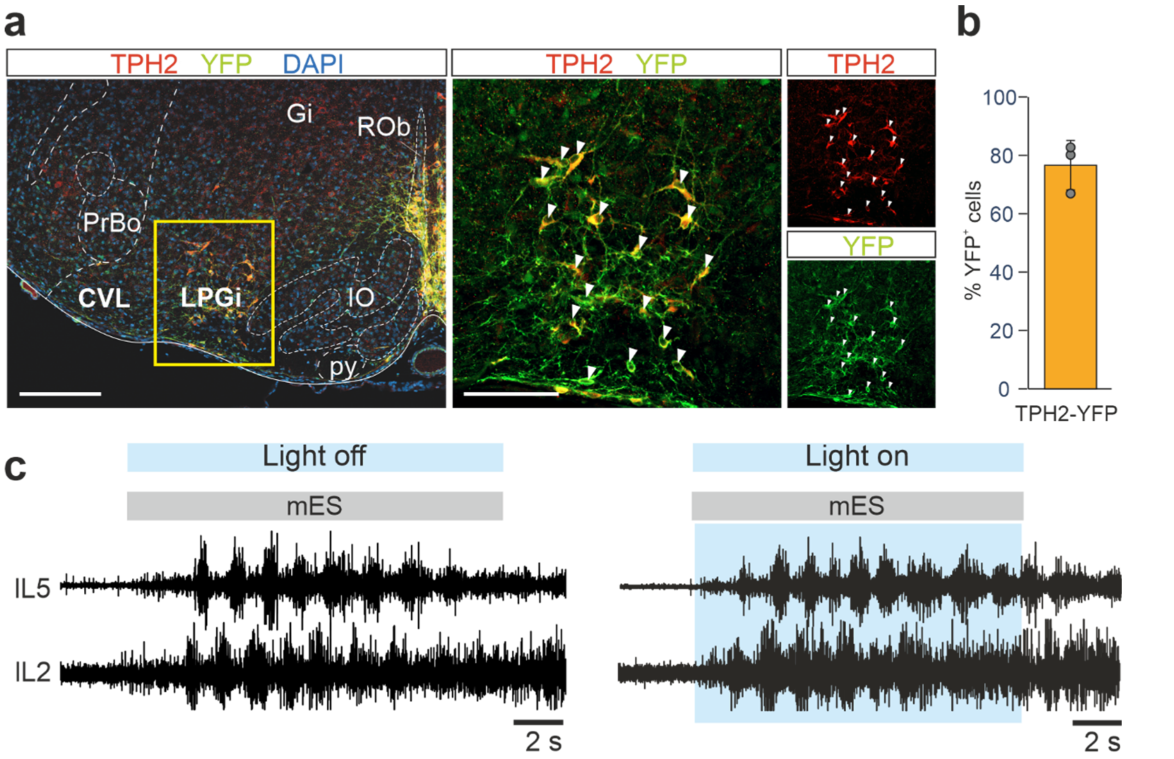

Supplementary Fig. 4 | Activation of SERT^{+} cells did not evoke a clear change in frequency of the mES-evoked locomotor-like activity. a, **b** *SERT^{Cre};R26R$^{ChR2\text{-}YFP}$* mouse line validation**.** **a** Transverse section of brainstem stained for TPH2^{+} and YFP^{+} cells at the level of rostral 12N. TPH2^{+} and YFP^{+} cells are colocalized. Abbreviation as in Fig. 1. Scale bars (in µm): left: 200; right: 100. **b** Quantification of the colocalization between TPH2^{+} and YFP^{+} cells. (n=15 sections from 3 animals). Grey dots indicate values for individual animals. Error bar indicates standard deviation. Source data are provided as a Source Data file. **c** mES-evoked locomotor activity with (right panel) and without (left panel) optogenetic activation of the SERT^{+} cells.

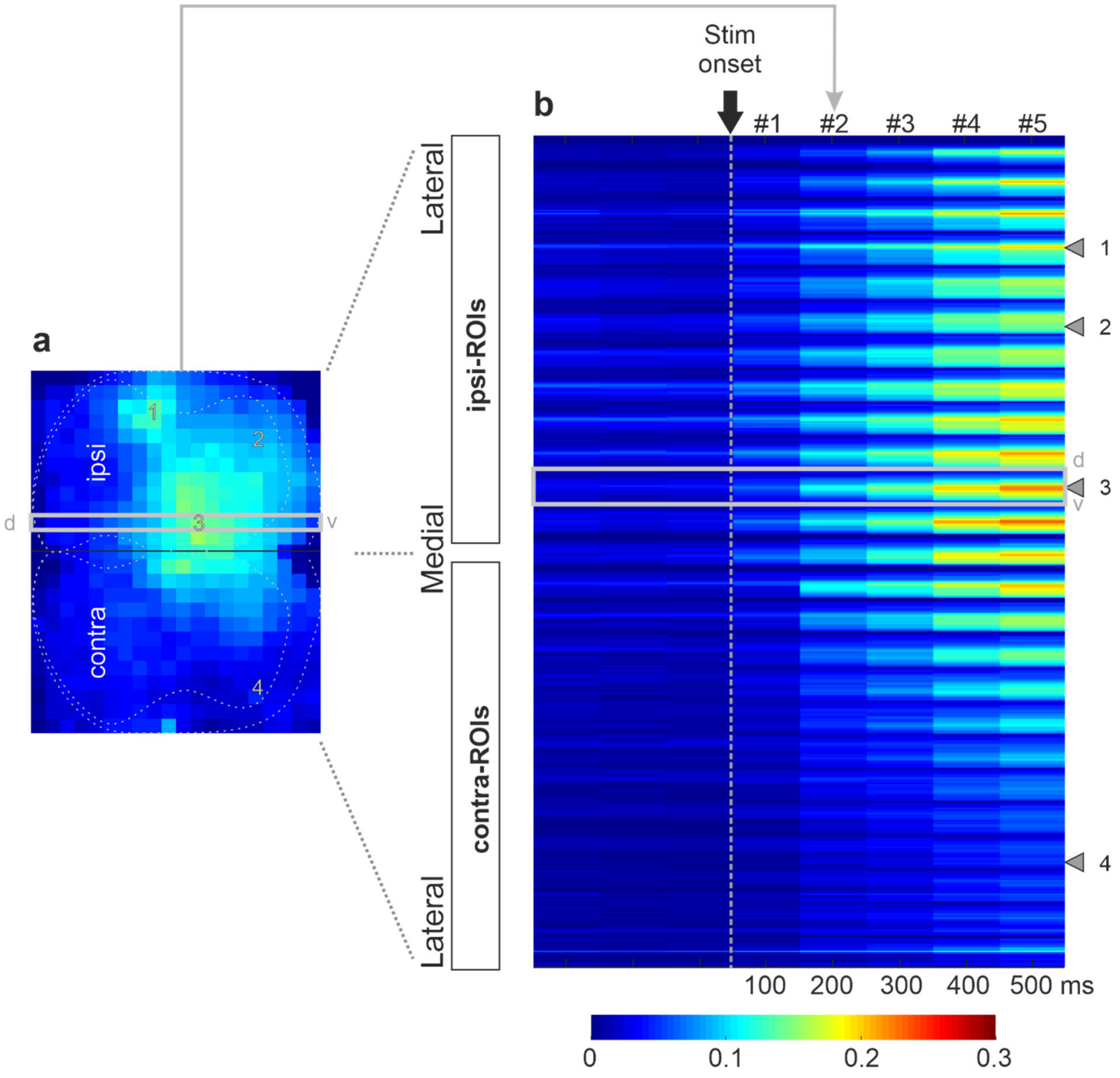

Supplementary Fig. 5 | Early onset of Ca^{2+} activity in the initiation phase. a Vglut2 Ca^{2+} activity 200 ms after stimulation onset. There is strong activation of grid-ROI 1 and 3. **b** Time-series with heatmap of activity of all grid-ROIs shown in (**a**) 300 ms before and the first 500 ms following the onset of stimulation. #1-4 indicates individual frames. One frame equals 100 ms. Each dorsal ('d')—ventral ('v') stretching row (illustrated by the gray box) in **a** is represented by a time-series column in **b**. In each column, the ROIs from the d-v stretching row in **a** are represented with dorsal upwards and ventral downwards in **b**.

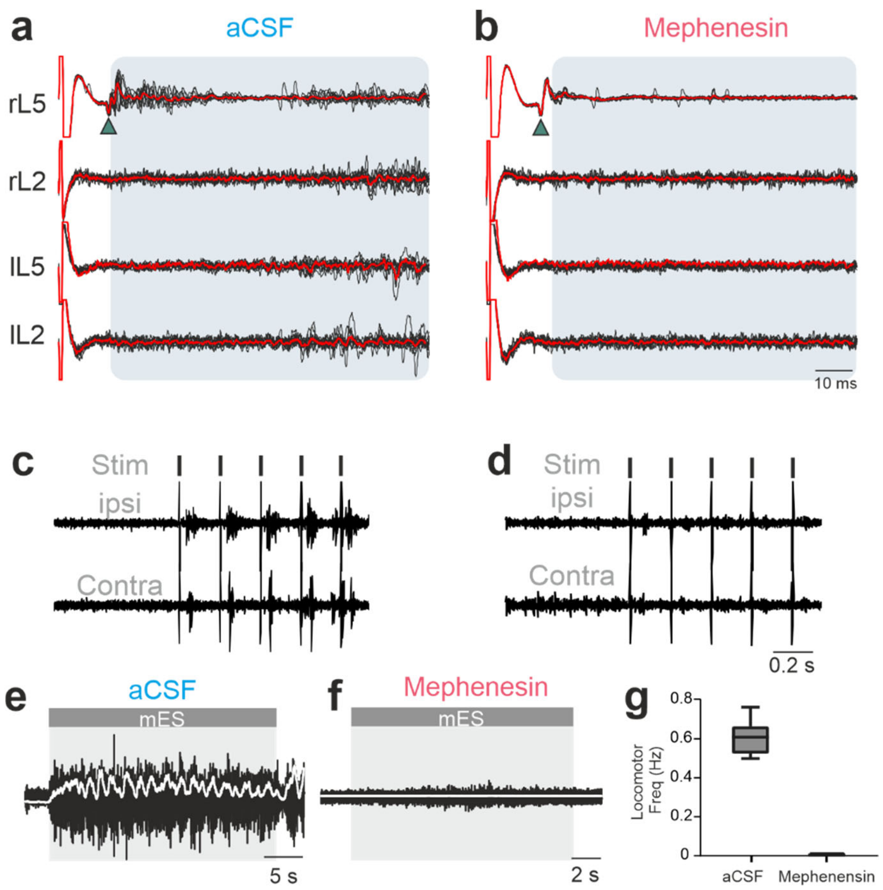

Supplementary Fig. 6 | Mephenesin attenuated or blocked polysynaptic transmission in the rodent spinal cord. a, b Mephenesin blocked the late-appearing polysynaptic responses to dorsal root stimulation. The right L5 dorsal root was stimulated with a single pulse while ventral roots (L2 and L5) were recorded on the right (r) and left (l) sides. The green triangles indicate the monosynaptic response in rL5 that is mostly unchanged in amplitude. (N=2 animals). **c, d** Polysynaptic responses in the ventral roots evoked by LPGi/CVL stimulation were blocked under mephenesin. **e-g** Locomotor activity evoked by LPGi/CVL stimulation was blocked under mephenesin. (N=12 animals). Box-whisker plot in 6g shows median (middle line), 25th, 75th percentile and maximal and minimal value. Source data are provided as a Source Data file.

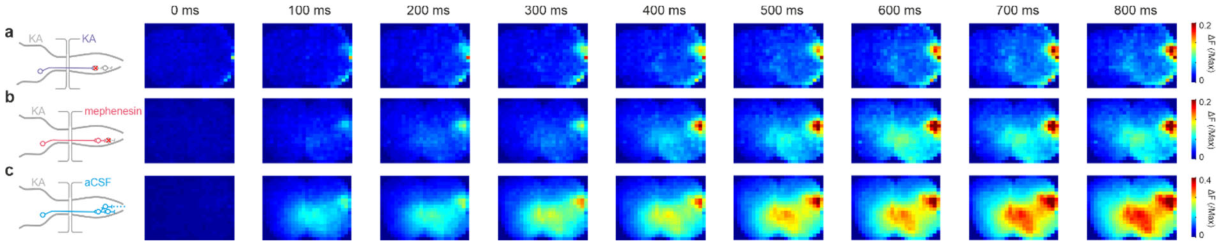

Supplementary Fig. 7 | Early activity in iEM. a-c Sequential activity maps in the early initiation phase after stimulation onset (up to 800 ms) under KA (**a**), mephenesin (**b**), and aCSF (**c**) conditions. The iEM is activated within the first frame of mapping (first 100 ms) after stimulation onset and maintained to be active under both mephenesin and aCSF conditions.

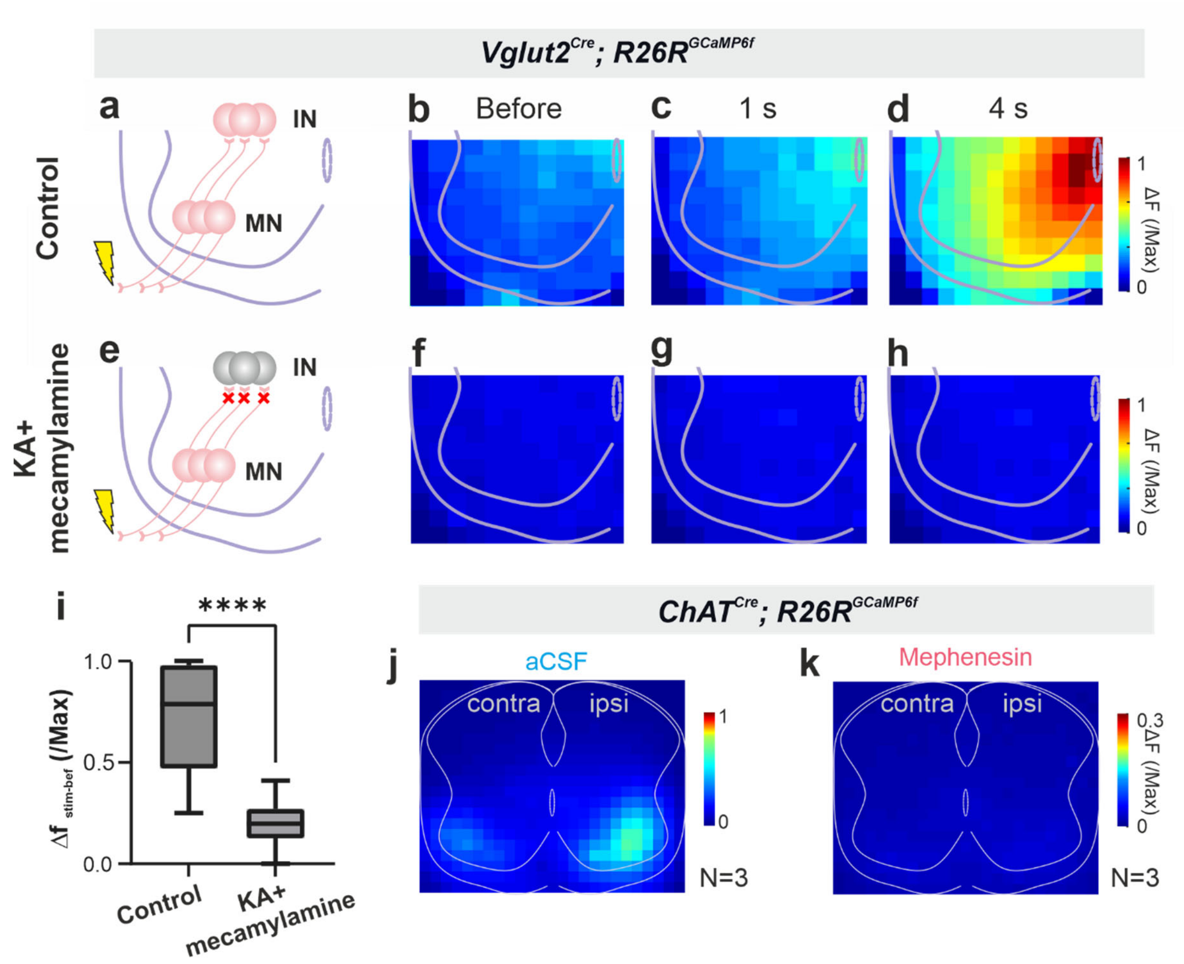

Supplementary Fig. 8 | Vglut2$^+$ Ca^{2+} activity during ventral root stimulation suggests that the prM activity originates predominantly from Vglut2 non-motor neurons. a-i Sequential activity maps from the ventral spinal cord before and during ventral root stimulation under control (**a-d**) and after blocking the effect on spinal circuits from motor neuron collaterals (**e-h**). The imaging is ipsilateral to the ventral root stimulation. There was a weak Vglut2$^+$ activity in the motor neuron area but an intense signal in the ventral spinal cord during stimulation in the control condition (normal aCSF). Both signals were strongly reduced after adding the glutamatergic receptor blocker KA and the nicotinic receptor blocker mecamylamine to the bath. **i** Quantification of the Vglut2 Ca^{2+} in the ventral cord in control and after KA + mecamylamine. The signal is strongly reduced in the ventral horn (n= 12 trials from 3 animals, paired t test – two sided, p=0.00000272). with little signal in the motor neuron area. Source data are provided as a Source Data file. **j**, **k** ChAT Ca^{2+} activity evoked by LPGi/CVL stimulation was blocked under mephenesin in *ChATCre; R26RGCaMP6f* mice (N=3 animals). Under aCSF perfusion, there was clear activity in ipsilateral clusters corresponding to the location of motor neurons, which is more ventrally located and in a smaller area than the prM area in the *Vglut2Cre; R26RGCaMP6f* mice. Box-whisker plot in 8i shows median (middle line), 25th, 75th percentile and maximal and minimal value. Source data are provided as a Source Data file.

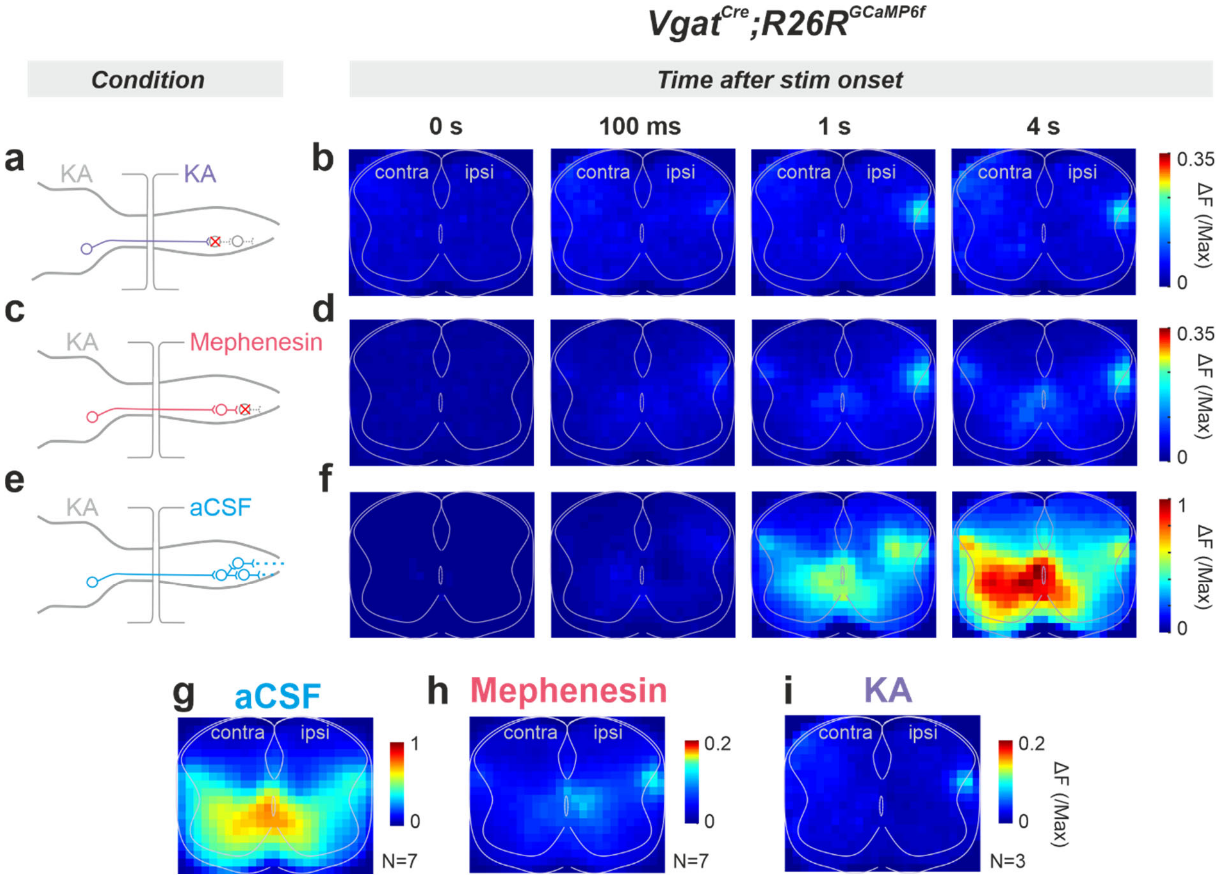

Supplementary Fig. 9 | Pharmacology of Vgat Ca^{2+} imaging in the initiating phase. a, **c**, **e** Schematics of the experiments and putative neuronal connection with each drug. **b**, **d**, **f** Activity maps at different time points after the stimulation, under KA (**a**, **b**), mephenesin (**c**, **d**), and aCSF conditions (**e**, **f**). See Results for detailed explanation. **g-i** Averaged maps of all preparations under the KA (**g**), mephenesin (**h**), and aCSF (**i**) conditions 4 s after stimulation onset. Source data are provided as a Source Data file.

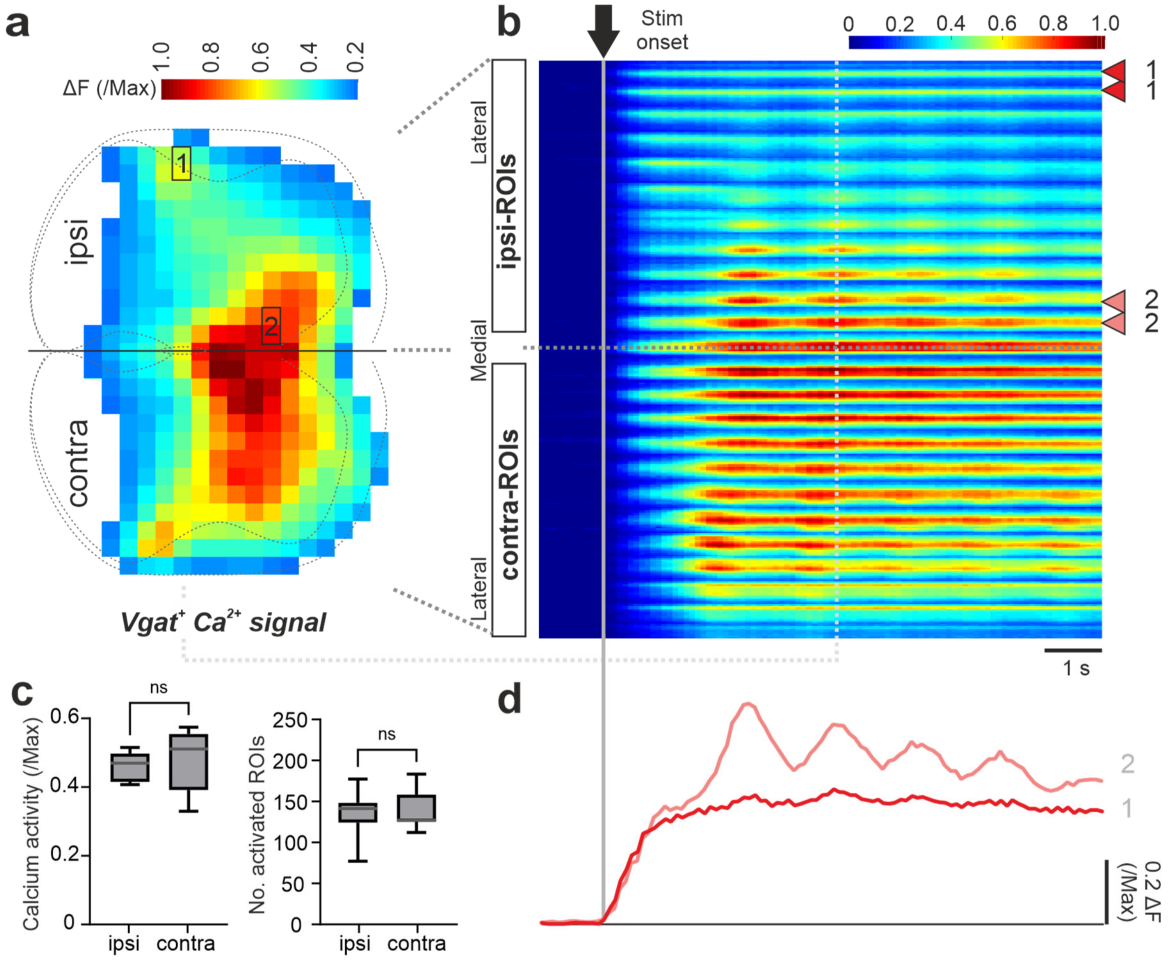

Supplementary Fig. 10 | Ca^{2+} activity of the $Vgat^{+}$ neurons during LPGi/CVL stimulation. **a** Activity map of the transverse section of the spinal cord 4 seconds after stimulation onset. Only the grid-ROIs with activity larger than the 0.2 maximal value were plotted. Specific areas were numbered as Areas 1-2, which contain two grid-ROIs respectively. **b** Heatmap of activity of individual grid-ROIs shown in (**a**) before and during stimulation. The numbered triangles correspond to the grid-ROIs in the numbered areas in d. Each row represents the Ca^{2+} activity for a grid-ROI across time. See Supplementary Fig. 5 for details. **c** The intensity of Ca^{2+} activity (left) and the number of activated ROIs (right) were not significantly different in the ipsi- and contra-lateral spinal cord (one-sided two-way repeated measure ANOVA - $p = 0.047$; *post-hoc* analysis - $p=0.52$ and 0.87 for Ca^{2+} activity and No. of activated ROIs, respectively, N=7 animals). Box-whisker plot shows median (middle line), 25th, 75th percentile and maximal and minimal value. Source data are provided as a Source Data file. **d** Example of Ca^{2+} traces for two different ROIs (1,2 in (**a**) and (**b**)) during stimulation.
