# Supplementary material for: Deconstructing the modular organization and real-time dynamics of mammalian spinal locomotor networks
Source: Nat Commun. 2023 Feb 16;14:873. doi: 10.1038/s41467-023-36587-w (PMC9935527; doi:10.1038/s41467-023-36587-w)
Supplement: Supplementary file 3 — Reporting Summary [file 41467_2023_36587_MOESM3_ESM.pdf]

nature portfolio

Corresponding author(s): Ole Kiehn
Last updated by author(s): Jan 6, 2023

Reporting Summary

Nature Portfolio wishes to improve the reproducibility of the work that we publish. This form provides structure for consistency and transparency in reporting. For further information on Nature Portfolio policies, see our Editorial Policies and the Editorial Policy Checklist.

Statistics

For all statistical analyses, confirm that the following items are present in the figure legend, table legend, main text, or Methods section.

n/a	Confirmed	
☐	☒	The exact sample size (n) for each experimental group/condition, given as a discrete number and unit of measurement
☐	☒	A statement on whether measurements were taken from distinct samples or whether the same sample was measured repeatedly
☐	☒	The statistical test(s) used AND whether they are one- or two-sided<br>*Only common tests should be described solely by name; describe more complex techniques in the Methods section.*
☐	☒	A description of all covariates tested
☐	☒	A description of any assumptions or corrections, such as tests of normality and adjustment for multiple comparisons
☐	☒	A full description of the statistical parameters including central tendency (e.g. means) or other basic estimates (e.g. regression coefficient) AND variation (e.g. standard deviation) or associated estimates of uncertainty (e.g. confidence intervals)
☐	☒	For null hypothesis testing, the test statistic (e.g. F, t, r) with confidence intervals, effect sizes, degrees of freedom and P value noted<br>*Give P values as exact values whenever suitable.*
☒	☐	For Bayesian analysis, information on the choice of priors and Markov chain Monte Carlo settings
☒	☐	For hierarchical and complex designs, identification of the appropriate level for tests and full reporting of outcomes
☒	☐	Estimates of effect sizes (e.g. Cohen's d, Pearson's r), indicating how they were calculated

Our web collection on statistics for biologists contains articles on many of the points above.

Software and code

Policy information about availability of computer code

Data collection	Electrophysilogical data were collected by pClamp (Clampex v.10, Molecular Devices); Clampfit v10.7; Molecular device. Calcium imaging data were collected by Camware (PCO) or HCImage (Hamamatsu). Anatomical imaging data were collected by Zen (v. 3.5, Zeiss).
Data analysis	Analysis of electrophysilogical data was done by SpinalCore, that is provided by Dr. Aharon Lev-Tov, and Dr. Yoav Mor. Analysis of calcium imaging data was done by ImageJ, Clampex, and MATLAB (R2018b). GraphPad (v.9, GraphPad Software). Anatomical imaging data were analyzed by ImageJ.

For manuscripts utilizing custom algorithms or software that are central to the research but not yet described in published literature, software must be made available to editors and reviewers. We strongly encourage code deposition in a community repository (e.g. GitHub). See the Nature Portfolio guidelines for submitting code & software for further information.

Data

Policy information about availability of data

All manuscripts must include a data availability statement. This statement should provide the following information, where applicable:

- Accession codes, unique identifiers, or web links for publicly available datasets
- A description of any restrictions on data availability
- For clinical datasets or third party data, please ensure that the statement adheres to our policy

The data generated in this study are available in the source data provided with this paper. Raw data files (i.e., videos and images) are not permanently deposited in an open access depository, but are available from the corresponding author upon request. Source data are provided with this paper. The Developing Mouse Brain Atlas (Allen Brain Atlas) was used as reference atlas for the brainstem.

Human research participants

Policy information about studies involving human research participants and Sex and Gender in Research.

Reporting on sex and gender	This is not a human research
Population characteristics	This is not a human research
Recruitment	This is not a human research
Ethics oversight	This is not a human research

Note that full information on the approval of the study protocol must also be provided in the manuscript.

Field-specific reporting

Please select the one below that is the best fit for your research. If you are not sure, read the appropriate sections before making your selection.

☒ Life sciences ☐ Behavioural & social sciences ☐ Ecological, evolutionary & environmental sciences

For a reference copy of the document with all sections, see nature.com/documents/nr-reporting-summary-flat.pdf

Life sciences study design

All studies must disclose on these points even when the disclosure is negative.

Sample size	We did not perform power analysis to determine necessary sample size. We used similar sample size as reported previously for anatomical and experiments of this type (Cregg et al. 2020; Nature Neuro; Bouvier et al. 2015 Cell). The magnitude of the observed effects indicate that the sample size is appropriate.
Data exclusions	No data were excluded from the analysis.
Replication	All experimental results were replicated in multiple runs of experiments with consistent results. Core elements including the electrical brain stem stimulation was replicated in two different sites: Kiehn lab at the University of Copenhagen and at the Karolinska Institute.
Randomization	Allocation of animals to the experimentally groups was done by picking random litter matter of 0-4 days old pups of of the right genotype or controls. Both sexes were used and all littermates were treated and kept equally before experiments.
Blinding	For the SERT-ChR2 experiments, the experimenters are blinded from the genotype. For Vglut2-ChR2 experiments, blinding was not possible since the phenotype to light stimulation is strong. For other experiments (ChAT-Tdtomato, calcium imaging experiments), blinding was not possible since (1), for conducting electrical stimulation, the experimenters have to actively search for the locomotor-effective spots, and/or (2) the imaging data from different strains is obviously different, and thus was noted by the experimenters.

Reporting for specific materials, systems and methods

We require information from authors about some types of materials, experimental systems and methods used in many studies. Here, indicate whether each material, system or method listed is relevant to your study. If you are not sure if a list item applies to your research, read the appropriate section before selecting a response.

Materials & experimental systems

n/a	Involved in the study	
☐	☒	Antibodies
☒	☐	Eukaryotic cell lines
☒	☐	Palaeontology and archaeology
☐	☒	Animals and other organisms
☒	☐	Clinical data
☒	☐	Dual use research of concern

Methods

n/a	Involved in the study	
☒	☐	ChIP-seq
☒	☐	Flow cytometry
☒	☐	MRI-based neuroimaging

Antibodies

Antibodies used	We used the following primary antibodies: chicken anti-GFP (polyclonal, Abcam, ab13970), rabbit anti-tdTomato (polyclonal, Takara, 632496), goat anti-CTB (polyclonal, List Biological Laboratories #703), mouse anti-TPH (Monoclonal, Merck-Millipore MAB5278) and rabbit anti-TPH2 (polyclonal, Merck-Millipore ABN60). Secondary antibodies (all from Invitrogen): Alexa-488 anti-chicken #A11039; Alexa-568 anti-rabbit #A10042; Alexa-555 anti-goat #A21432; Alexa-555 anti-mouse, #A21127.

Validation	The antibodies used have been validated in numerous publications and with Western/IHC on mouse samples as indicated on manufacturer's product page. The following antibodies are evalauetd in the antibody Register. AB_300798 (chicken-GFP); AB_2890005 rabbit-anti-tdTomato, :AB_10013220 (goat-anti-CTB)

Animals and other research organisms

Policy information about studies involving animals; ARRIVE guidelines recommended for reporting animal research, and Sex and Gender in Research

Laboratory animals	The following transgenic lines of mice of both sexes were used for breeding: Vglut2Cre (Borgius et al. 2010), VgatCre (Jackson Stock 016962), SERTCre (Jackson Stock 014554), ChATcre (Jackson Stock 006410), R26RGCaMP6f (Jackson Stock 030328), R26RTdtomato (Jackson Stock 007914), R26RYFP (Jackson Stock 006148), and R26RChR2-EYFP (Jackson Stock 012569). For experiments mice of both sexes aged 0-4 days were used. Mice were housed in standard cages 4-5 animals together in a 12/12 dark/light cycle at (housing temperature 23–24°C, 45–65% humidity).
Wild animals	No wild animal was used
Reporting on sex	This information was not collected. Presumably the data-set will contain a balanced distribution of either sex. .
Field-collected samples	No field-collected samples were used
Ethics oversight	All animal experiments and procedures were approved by the local ethical committee, Stockholm’s Norra Forsöksdjursnämnd and Dyreforsøgstilsynet in Denmark. These experiments were performed in accordance with European guidelines for the care and use of laboratory animals.

Note that full information on the approval of the study protocol must also be provided in the manuscript.
